# Emission of methane, carbon monoxide, carbon dioxide and short‐chain hydrocarbons from vegetation foliage under ultraviolet irradiation

**DOI:** 10.1111/pce.12489

**Published:** 2015-01-23

**Authors:** WESLEY T. FRASER, EMANUEL BLEI, STEPHEN C. FRY, MARK F. NEWMAN, DAVID S. REAY, KEITH A. SMITH, ANDY R. MCLEOD

**Affiliations:** ^1^ School of GeosciencesThe University of EdinburghEdinburghEH9 3FFUK; ^2^ The Edinburgh Cell Wall GroupInstitute of Molecular Plant SciencesThe University of EdinburghEdinburghEH9 3BFUK; ^3^ Royal Botanic GardenEdinburghEH3 5LRUK; ^4^ Present address: Department of Social SciencesOxford Brookes UniversityGipsy LaneOxfordOX3 0BPUK

**Keywords:** CH_4_, epidermal absorbance, flavonoids, lignin, pectin, UVA, UVB

## Abstract

The original report that plants emit methane (CH_4_) under aerobic conditions caused much debate and controversy. Critics questioned experimental techniques, possible mechanisms for CH_4_ production and the nature of estimating global emissions. Several studies have now confirmed that aerobic CH_4_ emissions can be detected from plant foliage but the extent of the phenomenon in plants and the precise mechanisms and precursors involved remain uncertain. In this study, we investigated the role of environmentally realistic levels of ultraviolet (UV) radiation in causing the emission of CH_4_ and other gases from foliage obtained from a wide variety of plant types. We related our measured emissions to the foliar content of methyl esters and lignin and to the epidermal UV absorbance of the species investigated. Our data demonstrate that the terrestrial vegetation foliage sampled did emit CH_4_, with a range in emissions of 0.6–31.8 ng CH_4_ g^−1^ leaf DW h^−1^, which compares favourably with the original reports of experimental work. In addition to CH_4_ emissions, our data show that carbon monoxide, ethene and propane are also emitted under UV stress but we detected no significant emissions of carbon dioxide or ethane.

## Abstract 2

This study examined the emission of methane and other gases from the foliage of a wide variety of plant species under environmentally realistic levels of ultraviolet radiation in order to investigate earlier controversial reports of aerobic methane emission from vegetation. Methane emissions ranged from 0.6–31.8 ng CH_4_ g^‐1^ leaf dry weight and were influenced by the foliar content of lignin and epidermal absorbance at 375 nm. Carbon monoxide, ethene and propane were also emitted under ultraviolet irradiation. The measured rates of methane emission corroborate the observations of earlier studies but do not suggest a need to revise an earlier estimate that UV‐driven methane emissions from foliage contribute less than 0.2% of global methane sources.

**Abbreviations**
AIR: alcohol‐insoluble residue;
ROS: reactive oxygen species
ME: methyl

## Introduction

Methane (CH_4_) is a naturally occurring gas in the Earth's atmosphere, currently at a global mean concentration of 1803 nL L^−1^, and is a known potent greenhouse gas with an atmospheric lifetime of 12.4 years and 100 year global warming potential that is 28 times that of carbon dioxide (CO_2_) (IPCC 2013). Consequently, small increases in CH_4_ concentration will result in a large increase in warming potential and natural and anthropogenic sources and sinks need to be identified and accurately quantified in order to calculate a global CH_4_ budget. Bousquet *et al*. (2006) demonstrated that global atmospheric CH_4_ content has almost tripled since the pre‐industrial period, with significant regional variability, most notably in the tropics. Regions of the southern hemisphere had relatively stable CH_4_ concentrations over the period 1984 to 2003, whereas regions of the northern hemisphere exhibited an overall decrease in concentration followed by a sharp rise at the end of the observation period. Concentrations in the tropics continued to be highly variable throughout and it may require careful study to understand the detail of their contribution to the global CH_4_ budget. The most recent review of three decades of global CH_4_ sources and sinks suggests that a rise in natural wetland and fossil fuel emissions probably accounts for the increase in global CH_4_ levels after 2006, with uncertainty about the relative contributions of these sources (Kirschke *et al*. 2013).

Initial work conducted by Keppler *et al*. (2006) showed that vegetation foliage emits CH_4_ under aerobic conditions and they suggested that it could make a significant contribution to atmospheric composition because of the vast areas of vegetation on the planet. Hitherto, CH_4_ emissions were assumed to be associated only with anaerobic processes. Based on a rudimentary upscaling from their laboratory‐based emission rates, Keppler *et al*. (2006) suggested that the global aerobic emission of CH_4_ from vegetation may be 62–236 Tg CH_4_ year^−1^, presenting an exciting but controversial stimulus to the research community. Subsequent studies into the global significance of possible plant‐derived CH_4_ emissions have suggested that this initial calculation of Keppler *et al*. (2006) may have been an overestimation. In particular, several workers questioned the original methodological approach, including Kirschbaum *et al*. (2006) who observed that it was inappropriate to assume that all net primary production contributes to foliar biomass that can generate CH_4_. They recalculated a global emission rate using alternative methods based on photosynthesis rate and leaf mass, which suggested a substantially lower emission rate of only 10–60 Tg CH_4_ year^−1^. Houweling *et al*. (2006) also re‐evaluated global CH_4_ emission rates using an atmospheric transport model and found an upper limit of 125 Tg CH_4_ year^−1^, but suggested a more plausible limit of ∼85 Tg CH_4_ year^−1^, values much closer to those of Keppler *et al*. (2006). They suggested that aerobic CH_4_ emissions from vegetation could account for up to 50% of the CH_4_ anomaly observed downwind of the Amazon basin by Frankenberg *et al*. (2005), who had compared CH_4_ data from satellite instruments with estimates derived from a global transport model combined with ground‐based source inventories. More recent studies showed that the satellite‐derived CH_4_ data were positively biased in tropical regions by spectroscopic interference by water vapour, but source inventories based on an updated data retrieval method still suggest substantial tropical CH_4_ emissions (Frankenberg *et al*. 2008). Houweling *et al*. (2006) suggested that they may have underestimated true global emissions from vegetation, as Keppler *et al*. (2006) only considered a limited number of species; a broader range of species may reveal a higher mean vegetation CH_4_ output.

Keppler *et al*. (2006) measured CH_4_ emissions not only from foliage, but also from purified structural components of plants, in particular pectin. Others (Keppler *et al*. 2008; McLeod *et al*. 2008; Vigano *et al*. 2008; Bruhn *et al*. 2009; Messenger *et al*. 2009) all confirmed pectin as a source, when subjected to ultraviolet radiation (UV: 280–400 nm). Methyl (Me) ester groups attached along the galacturonic acid backbone of the pectin polymer were suggested to be the most likely source of the CH_4_, liberated via a mechanism involving reactive oxygen species (ROS) as an intermediary step (Keppler *et al*. 2008; Messenger *et al*. 2009). Other components of foliage that have also been shown to be sources of CH_4_ under UV irradiation include lignin and cellulose (Vigano *et al*. 2008), leaf surface wax (Bruhn *et al*. 2014) and wood (Lee *et al*. 2012). Most recently, Wang *et al*. (2013) have reviewed non‐microbial CH_4_ production and concluded that it may occur in any organism when exposed to a range of environmental stresses.

Bloom *et al*. (2010) employed a UV climatology model to estimate global emissions of CH_4_ from foliar pectins, basing emission rates on those obtained from commercial pectin (McLeod *et al*. 2008; Messenger *et al*. 2009) and assuming a leaf pectin content of 5% DW. Their global CH_4_ emission value was much lower than previous estimates at 0.2–1.0 Tg CH_4_ year^−1^, approximately 0.2% of the total global CH_4_ source. However, their calculations were not based on emission rates from plant tissue and Bruhn *et al*. (2012) have suggested that such upscaling calculations are inadequate owing to uncertainties about the effect of environmental factors stimulating emissions, genotypic responses and the range of other possible CH_4_ precursors. Consequently, there is a strong case for establishing CH_4_ emission rates using actual plant leaves exposed to ambient levels of UV radiation.

Previous efforts to detect and quantify CH_4_ emissions from plants have either used chambers or vials constructed from material [e.g. ‘Perspex’ (polymethylmethacrylate), polycarbonate or glass] that does not transmit any UV‐B and only some UV‐A radiation (see McLeod *et al*. 2008) or have used illumination that excluded UV wavelengths during emission measurements (Keppler *et al*. 2006; Dueck *et al*. 2007; Beerling *et al*. 2008; Qaderi & Reid 2009). We therefore undertook a series of measurements on a range of plant species, using temperature‐controlled chambers with a UV‐transmitting quartz window and levels of spectrally weighted UV irradiance within the ambient range. As plastics and rubbers are hydrocarbon‐derived and may potentially release CH_4_ or other hydrocarbons into the measurement chamber under UV irradiation conditions (Stephenson *et al*. 1961; Fraser *et al*. unpublished data), we avoided their use in our experimental system. In addition, we chose to examine detached leaves in order to avoid the potential confounding effect of dissolution of soil‐derived CH_4_ in the transpiration stream. The primary objective of our study was to determine whether naturally relevant levels of UV radiation cause an emission of CH_4_, carbon monoxide (CO), carbon dioxide (CO_2_) and a range of short‐chain hydrocarbons [ethene (C_2_H_4_), ethane (C_2_H_6_), propane (C_3_H_8_)] from terrestrial vegetation foliage, sampled from a range of plant types. By using a novel experimental system that avoided some previous criticisms, we aimed to clarify earlier reports that CH_4_ is generated *in situ* in the leaves of terrestrial vegetation. We also examined the lignin and Me ester contents of our samples and compared our emission rates with retrospective measurements of the epidermal UV absorbance of leaves from the sampled plant specimens.

## Materials and Methods

All chemical reagents were obtained from the Sigma‐Aldrich Chemical Company, (Poole, Dorset, UK) and all gases used for GC calibration from BOC Special Gases (Guildford, Surrey, UK), unless otherwise mentioned.

### Leaf samples

**Figure 1:**
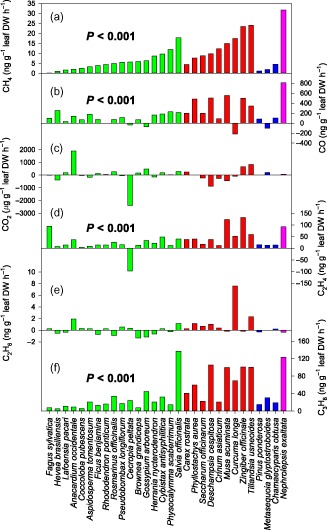
Net UV‐induced gaseous emissions from terrestrial plant leaves of 30 species irradiated with 7.1 W m^−2^ (CH_4_‐weighted) ultraviolet radiation (280–400 nm) from Q‐Panel UV313 lamps filtered with 125 *μ* m cellulose diacetate at 25 °C. 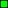 Dicotyledoneae; 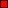 Monocotyledoneae; 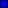 Pinopsida; 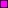 Polypodiopsida. (a) CH_4_; (b) CO; (c) CO_2_; (d) C_2_ H_4_; (e) C_2_ H_6_; (f) C_3_ H_8_. Vertical bars represent single replicate measurements of each species (*n* = 1). Probability values indicate a significant difference between pooled values of irradiated and non‐irradiated leaves from all species when tested by the Mann–Whitney test (*n* = 30).

Thirty species for study were selected from a range of temperate and tropical species of trees, shrubs, herbs, grasses, a sedge and a fern from the collection of the Royal Botanic Garden, Edinburgh (RBGE), located at UK National Grid Reference NT 247754 (55.97° N, 3.21° W). Source plants were grown either under horticultural glass or outside, depending on the particular growing needs of each species. Detached leaf samples were collected daily between 0800 and 0900 BST during May–June 2011 before experimental irradiation in the laboratory, selecting from the youngest fully expanded leaves on the edge of the canopy on a single plant. For all experiments, the time interval between collection of leaves and the commencement of each experiment was less than 3 h. Leaves were randomly allocated to either an un‐irradiated or UV‐irradiated chamber in order to cover the chamber surface area without overlapping leaves. The area of leaves was scanned and measured using ImageJ software (Abramoff *et al*. 2004). All leaves were oven dried at 50 °C for 48 h after experimentation to obtain dry mass. The species investigated are shown in Fig. 1 and details of species authorities and growing site are shown in Supporting Information Table S1.

### Experimental chambers

Four bespoke circular chambers of ∼280 mL volume (range of individual chambers: 274–283 mL) were cut from single aluminium blocks and each fitted with a series of gas ports around the perimeter. Quartz glass lids were used to allow UV wavelength radiation through to the enclosed leaves and sealed airtight using Krytox grease (Du Pont, Wilmington, DE, USA). A single 100 mL glass syringe was attached to one of the gas ports to act as an air reservoir during the experiment. Once sealed airtight with the leaf inside, the chambers were sampled twice (2 × 20 mL) before the beginning of an experiment, and twice (2 × 20 mL) after the completion of an experiment. The presence of the 100 mL reservoir syringe enabled chamber air to be sampled without any adverse pressure conditions drawing additional gas out of the leaf tissue. The base of each chamber contained a coolant reservoir for temperature control. Internal chamber temperature was maintained using a Thermo‐Haake Dynamax 7000 fast‐response chiller unit (Thermo Fisher Scientific, Karlsruhe, Germany) circulating a 50:50 mix of ethylene glycol : water through the base of the chambers. Chamber temperature was maintained at 25 °C throughout each experiment and laboratory air was also held at 25 °C by an air conditioning system in order to prevent condensation forming on the inside of the chambers.

### Gas quantification

Quantification of CH_4_, CO, CO_2_, C_2_H_4_, C_2_H_6_ and C_3_H_8_ emissions released from leaves was performed using a Varian GC‐450 gas chromatograph (GC) (Varian Inc., Palo Alto, CA, USA), fitted with a Hayesep‐Q column, running a flame ionization detector (FID) held at 290 °C. The carrier gas was N_2_ at a constant flow rate of 40 mL min^−1^. Detection of CO and CO_2_ was made possible by a methanizer (Varian Inc.) fitted between the column and FID. Analytical runs lasted for 11 min, comprising an initial isothermal step held at 50 °C for 2 min, followed by a constant temperature increase of 20 °C min^−1^ to a maximum temperature of 180 °C, which was then held for the remainder of the run. 20 mL samples were manually injected through a 2 mL sampling loop; an automated 0.1 min delay ensured pressure equilibration within the sampling loop before proceeding to the column. In order to preserve the Hayesep‐Q column and to remove any potential interference from water vapour in the gas samples, a Nafion filter (Brunswick Ltd., Hove, UK) was installed prior to the injection port. Using a counter‐flow of Zero Grade air (BOC Special Gases) at a flow rate of 200 mL min^−1^, >90% of moisture in the sample was removed. Daily calibration of GC response was performed using a dilution series of a known 7‐gas standard (BOC Special Gases) of the following composition: CO, 20.6 μL L^−1^; CO_2_, 1518 μL L^−1^; CH_4_, 3.2 μL L^−1^; C_2_H_4_, 19.5 μL L^−1^; C_2_H_6_, 19.3 μL L^−1^; C_3_H_8_, 10.0 μL L^−1^. The balance of the gas‐mix was N_2_. Dilutions of 50 and 25% were made from this standard using Zero Grade N_2_ (BOC Special Gases). Operation of the GC and peak integration were performed by Galaxy Chromatography Software Ver. 1.9 (Varian Inc., Walnut Creek, CA, USA). Possible CH_4_ production in empty chambers was checked under UV irradiation at intervals before, during and after this series of experiments and none was detected.

Each plant species was irradiated in a single experiment of 4 h duration using one pair of chambers; one chamber was exposed to UV radiation using only a cellulose diacetate (CA) film (125 *μ* m; Courtaulds Speciality Plastics, Derby, UK) to remove lamp emissions below 290 nm, including UV‐C wavelengths (<280 nm). A second chamber acted as a control with a CA‐filter plus an additional filter of 0.06 mm UV‐opaque polyester [Courtgard’ (CG) CPFilms Inc., Martinsville, VA, USA] placed on top of the chamber beneath the CA filter to block out UV‐B and most UV‐A wavelengths (<380 nm). Examples of the spectrum of CA‐filtered lamps and the transmission spectra of CA and CG filters are provided by McLeod *et al*. (2008). Net UV‐driven gas exchange was determined by subtracting the concentration change over the experimental period in the un‐irradiated control chamber from the concentration change in the UV‐irradiated chamber.

### Control of UV irradiance

UV irradiance was maintained at a constant level throughout using a dynamic feedback loop controlled by PC‐based software. An array of 12 40W Q‐Panel 313‐EL fluorescent UV lamps (The Q‐Panel Company, Cleveland, OH, USA) was suspended on a height‐adjustable frame above the experimental chambers, capable of providing irradiation across the UV‐B (280–315 nm) to UV‐A (315–400 nm) spectrum (see Supporting Information Fig. S1). Irradiation was always performed at the same lamp height of 0.42 m and lamps were adjusted by a phase‐angle dimming system operated at mains electrical frequency of 50 Hz and controlled to maintain a constant irradiance as measured by a broad‐band sensor (Model PMA2102; Solar Light Inc., Glenside, PA, USA).

The spectral irradiance of the system and calibration of the broad‐band sensor were determined using a double monochromator spectroradiometer (SR991‐PC, Macam Photometrics, Livingston, West Lothian, UK) calibrated against a tungsten and deuterium lamp traceable to National Physical Laboratory Standards (SR903, Macam Photometrics, Livingston, West Lothian, UK). The level of photosynthetically active radiation (PAR; 400–700 nm) and UV irradiance calculated using a range of common spectral weighting functions are given in Table 1. One level of irradiance was selected for screening all species, set at 7.1 W m^−2^ (CH_4_‐weighted irradiance), which McLeod *et al*. (2008) reported to be lower than modelled low‐latitude peak irradiances (that exceed 11 W m^−2^ on the CH_4_‐weighted irradiance scale) and the highest global irradiance, measured at Cuzco, Peru which exceeded 30 W m^−2^ CH_4_‐weighted UV.

**Table 1:** Photosynthetically active radiation (PAR) and ultraviolet irradiances during 4 h exposures to Q‐Panel 313 fluorescent lamps filtered with 125 *μ* m cellulose diacetate

Irradiance (W m^−2^)											
Total UV (280–400 nm)	UV‐A (315–400 nm)	UV‐B (280–315 nm)	PAR (400–700 nm)	CH_4_	CIE	GEN (G)	GEN (T)	PG	DNA	QUT	FLAV
11.6	6.2	5.4	2.6	7.1	1.4	2.5	2.6	2.3	2.48	2.89	3.25

UV irradiances calculated using a range of common spectral weighting functions: CH_4_, idealized spectral weighting function for CH_4_ production that decays one decade in 80 nm (McLeod *et al*. 2008); CIE, weighted with the Commission Internationale de l'Eclairage (CIE) erythemal action spectrum (McKinlay & Diffey 1987; Webb *et al*. 2011); GEN (G), weighted with a mathematical function (Green *et al*. 1974) of the general plant action spectrum (Caldwell 1971); GEN (T), weighted with a mathematical function (Thimijan *et al*. 1978) of the general plant action spectrum (Caldwell 1971); PG, weighted with a the plant growth function of Flint & Caldwell (2003); DNA, weighted with the DNA damage action spectrum (Setlow 1974); QUT, weighted with the pyridine dimer action spectrum (Quaite *et al*. 1992); FLAV, weighted with the function for accumulation of the flavonol conjugate mesembryanthin in *Mesembryanthemum crystallinum* (Ibdah *et al*. 2002).

### Preparation of alcohol‐insoluble residue

Approximately 1 g of fresh frozen sample of plant material was cooled with liquid nitrogen in a ceramic mortar and ground to a fine powder with a pestle, then 0.5 mL water, 5 mL methanol and 0.5 mL 90% formic acid were added before re‐grinding for 5–10 min to a smooth paste. If necessary a further volume of methanol was added in order to aid mixing. The paste was mixed with 5 mL chloroform and then centrifuged (1.7 × 10^3^ g, 5 min). The solid residue was re‐suspended in 80% ethanol, centrifuged and the supernatant discarded. The process was repeated until the supernatant was colourless. The residue was then washed with acetone until the supernatant was colourless and dried in air or under a vacuum to provide alcohol‐insoluble residue (AIR) for subsequent analyses.

### Assays for leaf lignin and leaf methyl ester content

Leaf lignin content was determined by a modification of the method of Johnson *et al*. (1961). One mL of acetyl bromide/acetic acid (1:3 v/v) was added to a sample of leaf AIR (10 mg), vigorously shaken, incubated at 70 °C for 30 min and cooled to 15 °C in a water bath. After 5 min, 100 *μ*L aliquots were mixed with 90 *μ*L 2 m NaOH followed by 0.5 mL glacial acetic acid. After mixing, 20 *μ*L 7.5 m NH_2_OH.HCl was added and the solution made up to exactly 8 mL with glacial acetic acid. Absorbance (280 nm) was read in triplicate with a Cary 50 UV/visible spectrophotometer with Cary WinUV software (Varian Inc., Victoria, Australia).

The Me ester content of leaf AIR was determined by a modification of the method of Fry (1994). Between 1 and 10 mg AIR was treated with 0.95 mL of 42 mm KOH at 25 °C for 60 min; the saponification products were neutralized and then assayed for methanol by incubation at 25 °C for 40 min with *Pichia pastoris* alcohol oxidase (final concentration 6.3 U mL^–1^; specific activity 10–40 units per mg protein; Sigma‐Aldrich product A2404) and ‘type II’ horseradish peroxidase (16 U mL^–1^, 150–250 pyrogallin units mg^−1^ solid; Sigma‐Aldrich product P8250) in 85 mm phosphate (K^+^) buffer (pH 7.7) containing 1.6 mm aminoantipyrene and 9.4 mm phenol. The coloured product was quantified from its absorbance at 546 nm.

### Optical measurements of leaf epidermal phenolic content

In order to evaluate the role of UV radiation in CH_4_ emissions further, we subsequently performed optical measurements of epidermal phenolic content of the leaves of our specimen plants. Leaves were selected on 15–16 July 2013 at the same positions and on the same plants as were used for investigating CH_4_ emissions in 2011 and measurements performed *in vivo* with a Dualex Flav 4.5 (Force‐A, Orsay, France). The Dualex instrument measures the excitation of chlorophyll in the mesophyll using both UV (375 nm) and red light (650 nm). The UV radiation is highly absorbed in the epidermis, predominantly by flavonoids, and by comparing the fluorescence induced by the UV and red wavelengths, the epidermal absorbance can be determined (Goulas *et al*. 2004). Twenty replicate readings were made within 2 h of solar noon on the upper (adaxial) surface only, as this surface was relevant to the experimental UV irradiation applied during measurement of CH_4_ emissions.

### Statistical analysis

Data for CH_4_ and other gas emission rates, lignin content and epidermal absorbance (*A*_375_) were not normally distributed, even after transformation, so non‐parametric statistical analyses were performed. As each species was investigated in a single experiment using an un‐replicated pair of chambers (one UV‐irradiated and one un‐irradiated control), this only allowed statistical analysis for emissions from all pooled samples but precluded analysis for differences between species. The effect of UV irradiance on emissions was tested separately for each gas across all 30 plant species by the Mann–Whitney test. Relationships between experimental CH_4_ emission rate and lignin content, Me ester content and *A*_375_ were investigated by correlation analysis using Spearman's rank correlation (Sokal & Rohlf 1995). Differences in the CH_4_ emissions rate between groups of species based upon their systematic classification in the plant kingdom and structure/functional type (tree, shrub, herb, grass, sedge, fern) were performed using the Kruskal–Wallis anova on ranks with the Dunn's method for paired comparisons between groups. All analyses were completed using SigmaPlot version 12.3 software (Systat Software Inc., San Jose, CA, USA).

## Results

**Figure 2:**
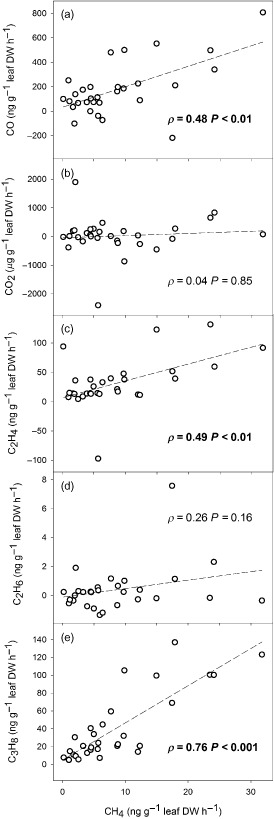
Correspondence plots showing the relationship of UV‐induced CH_4_ emissions with simultaneous emissions of (a) CO; (b) CO_2_; (c) C_2_ H_4_; (d) C_2_ H_6_; (e) C_3_ H_8_, with Spearman's correlation coefficients (*ρ*). Probability (*P*) values in bold indicate significant correlations between variables. Hatched lines indicate the linear trend.

The range of species demonstrated a broad spread of emission rates across the six gases measured (Fig. 1, Supporting Information Table S1). CO, CH_4_, C_2_H_4_ and C_3_H_8_ net emission rates from leaves were all enhanced under UV radiation and significantly different from equivalent leaves not exposed to UV radiation when tested across all species by the Mann–Whitney test (*P* < 0.001). There was no significant net UV‐induced emission of CO_2_ or C_2_H_6_ from the foliage sampled. Comparison of emissions of the different gases (Fig. 2) revealed that positive relationships existed between CH_4_ and other gas emissions. If a plant was a high emitter of CH_4_, it was also likely to show high emission rates for CO, C_2_H_4_ and/or C_3_H_8_, but there was no correlation between CH_4_ emissions and those of CO_2_ and C_2_H_6_.

**Figure 3:**
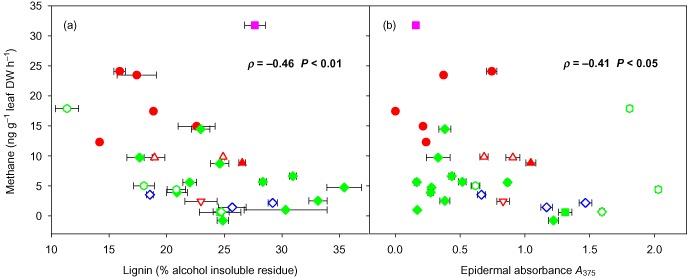
Net UV‐induced CH_4_ emissions from plant leaves irradiated with 7.1 W m^−2^ (CH_4_‐weighted) ultraviolet radiation (280–400 nm) plotted against (a) mean foliar lignin content (*n* = 3) and (b) mean epidermal absorbance at 375 nm (*n* = 20). Error bars (where visible) show ± standard error. Closed symbols are plants grown inside a glasshouse, open symbols are plants grown outside: ▲ grasses, ◆ trees, ● herbs, 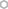 shrubs, ■ fern, ▼ sedge. Colours as in Fig. 1. Values show Spearman's correlation coefficients (*ρ*) with their significant probability values (*P*).

Leaf Me ester content fell within the range 21–480 *μ*mol g^−1^ AIR but showed no significant correlation with CH_4_ or other gas emission rates (data not shown). However, lignin content (Fig. 3a) and *A*_375_ (Fig. 3b) both showed a significant inverse correlation with CH_4_ emission rate across species with higher rates of emission corresponding to lower content of lignin and lower epidermal absorbance at 375 nm. There was no significant correlation between lignin content and *A*_375_.

**Figure 4:**
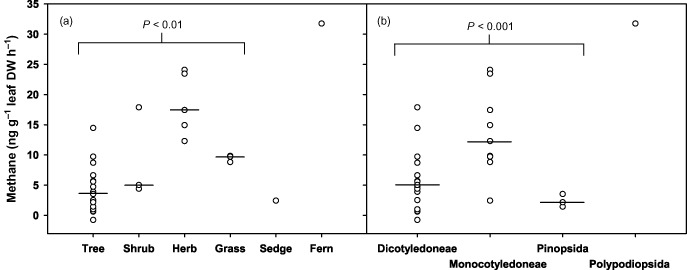
Net UV‐induced CH_4_ emissions from 30 species of plants grouped by (a) structural/functional type: trees (*n* = 17), shrubs (*n* = 3), herbs (*n* = 5), grasses (*n* = 3), sedge (*n* = 1), fern (*n* = 1); (b) on a taxonomic basis: Dicotyledoneae (*n* = 17), Monocotyledoneae (*n* = 9), Pinopsida (*n* = 3), Polypodiopsida (*n* = 1). Horizontal bars indicate the median of each group (where *n* > 1). Probability (*P*) values indicate significant differences between the groups within brackets when tested by the Kruskal–Wallis anova on ranks.

The CH_4_ emission rates of each species were also examined in ecologically relevant groups based on their structure/functional type (Fig. 4a) and their systematic classification (Fig. 4b). There were insufficient replicates within some groups [sedge (*n* = 1), fern/Polypodiopsida (*n* = 1)] for them to be analysed statistically. However, an anova on ranks by the Kruskal–Wallis method indicated that the remaining groups showed overall significant differences between the groups (tree, shrub, herb, grass: *P* < 0.01) and (dicotyledons, monocotyledons, Pinopsida: *P* < 0.001). Pairwise comparisons by the Dunn's method indicated that herbs had significantly higher CH_4_ emissions than trees (*P* < 0.001) and monocotyledons had significantly higher CH_4_ emissions than both dicotyledons and Pinopsida (*P* < 0.05).

## Discussion

### Methane emissions

The presence of CH_4_ in the suite of trace gas emissions corroborates the early finding of Keppler *et al*. (2006) of CH_4_ generation under aerobic conditions. While the level of replication in our study precludes conclusions about differences between individual species, we detected a net production of CH_4_ (range 0.61–31.76 ng CH_4_ g^−1^ leaf DW h^−1^) from the leaves tested and our analysis clearly demonstrates that UV radiation can produce a net CH_4_ emission from terrestrial vegetation foliage. The original work of Keppler *et al*. (2006) reported emissions of 1.1–21.6 ng CH_4_ g^−1^ leaf DW h^−1^ in sunlight from fresh detached leaves of a range of species. This is consistent with our results here, given the different source material, growing conditions and nature of UV exposure. The glass vials used with detached leaves by Keppler *et al*. (2006) did not transmit UV‐B radiation and only some UV‐A radiation, which may explain why we observed some higher emission rates in four of the five species common to both studies.

The use of detached leaves limited any potential contribution of CH_4_ dissolved in the transpiration stream to the observed emissions but raised questions about the effect of leaf excision on our observations. Un‐irradiated leaves were appropriately used as controls in all experiments to estimate net UV‐driven emissions but leaf excision itself would result in changes to stomatal water relations. Leaf excision has variable effects on stomatal conductance between species and between plants grown inside a glasshouse or outside (Powles *et al*. 2006) but eventually induces stomatal closure. This would influence any instantaneous measurement of CH_4_ emissions originating within the leaf. Stomatal conductance has been observed to approach a minimum value ‘close to zero’ in excised leaves only after 2–4 h by Powles *et al*. (2006). Consequently, we believe that over the 4 h experimental period, an equilibrium would be reached between the CH_4_ concentration in the sub‐stomatal cavity and the chamber volume and our observations will include any CH_4_ generated within the leaf.

Pectins have been suggested as an important source of aerobic CH_4_ production in leaves (Keppler *et al*. 2006; McLeod *et al*. 2008; Vigano *et al*. 2008; Bruhn *et al*. 2009; Messenger *et al*. 2009), with their Me groups identified as the origin by deuterium labelling studies (Keppler *et al*. 2008). Typically, 50–75% of the galacturonic acid residues in pectins carry one O‐bonded Me ester group and rhamnose residues (which can be 10% of pectin DW) have one C‐bonded Me group (Buchanan *et al*. 2000). Messenger *et al*. (2009) observed that pure sugars (including galacturonic acid and rhamnose) and their methyl esters do not appreciably absorb UV between 280 and 400 nm. They concluded that UV radiation does not act directly on methyl groups but via another UV‐absorbing ‘photosensitizer’ that generates ROS (principally the hydroxyl radical). As we observed no relationship between the Me ester content of leaves and their CH_4_ emission rate, we infer that not all Me groups in pectins or other molecules and their adjacent photosensitizers were irradiated or that other compounds contribute to the CH_4_ emission. Other compounds that contain Me groups include lignin (one or two O‐bonded Me ether groups on most monolignol units of the lignin polymer), chlorophyll *a* (one Me ester and 10 C‐bonded Me groups), chlorophyll *b* (one Me ester and nine C‐bonded Me groups), carotenoids (carotenes and xanthophylls: typically ∼10 C‐bonded Me groups), triglycerides (oil reserves: three C‐bonded Me groups), phospholipids (two C‐bonded Me groups plus an additional three N‐linked Me groups in the choline of the major phospholipid: phosphatidylcholine), amino acids [leucine, isoleucine and valine (two C‐bonded Me groups), threonine (one C‐bonded Me group) and methionine (one Me‐thioether group)], cutin and suberin (a few C‐bonded Me groups) and epicuticular waxes (C‐bonded Me groups; Buchanan *et al*. 2000).

Vigano *et al*. (2008) reported that UV irradiation of lignin (which contains Me groups) and cellulose (which does not) produces CH_4_, but at high UV irradiances using unfiltered lamps. Bruhn *et al*. (2014) have reported CH_4_ emissions from UV irradiation of epicuticular wax of *Brassica oleracea* L., and most recently, Althoff *et al*. (2014) have proposed an abiotic chemical mechanism for CH_4_ formation from methionine in living organisms.

Notably, we observed a significant inverse correlation of CH_4_ emission with foliar lignin content. Our lignin analysis measured methanol‐insoluble phenolic compounds, predominantly lignin but also including polysaccharide‐esterified ferulic acid and related phenolic acids, and these may therefore be shielding other source compounds from irradiation, as was observed for *ortho*‐coumaric acid by Messenger *et al*. (2009). We also observed an inverse correlation with epidermal UV absorption (*A*_375_) and both these relationships imply that UV irradiation penetrates beyond the epidermal layer to influence CH_4_ formation within the leaf. Bruhn *et al*. (2012) suggested that UV radiation does not penetrate beyond the epidermis, based upon the observations of Cen & Bornman (1993) and Liakoura *et al*. (2003). However, Day *et al*. (1993) reported that the epidermis can behave as a non‐uniform filter in some plants and that a considerable proportion of UV‐B radiation penetrates into the leaf mesophyll. Our observations of a correlation between epidermal UV absorbance and UV‐driven methane production across species are consistent with the suggestion that UV radiation can penetrate beyond the epidermal layer.

Bloom *et al*. (2010) estimated the global significance of UV‐driven CH_4_ emissions from foliage, based on laboratory measurements of emissions from plant pectins. They assumed a constant CH_4_ emission rate of 3.09 × 10^−11^ kg CH_4_ kg^−1^ pectin per unit irradiation (J m^−2^) and a foliar pectin content of 5%. This would produce a value of 39 ng CH_4_ g^−1^ leaf DW h^−1^ during our experimental exposures of 7.1 W m^−2^ CH_4_‐weighted UV. As this just exceeds the highest CH_4_ emission rate measured during our experiments, we consider it unnecessary to revise their suggestion that UV‐driven CH_4_ emissions from foliage contribute less than 0.2% of total global CH_4_ sources.

### Emissions of carbon monoxide, carbon dioxide and short‐chain hydrocarbons

Emission of CO from plant tissues has previously been reported by Zimmerman *et al*. (1978), Scharffe *et al*. (1990), Derendorp *et al*. (2011), Lee *et al*. (2012) and most recently by Bruhn *et al*. (2013). The latter study reported CO emission under natural sunlight from six plant species within the range of 965–2396 nmol CO m^−2^ h^−1^. Our range of CO emissions expressed per unit leaf area (from Supporting Information Table S1) extends up to 1696 nmol CO m^−2^ h^−1^, which falls within the same range. We detected no significant emissions of CO_2_ despite reports by McLeod *et al*. (2008) that UV irradiation of dry pectin produced CO_2_ emissions much higher than those of CH_4_. However, this current study used living leaf material and the UV irradiation included 2.6 W m^−2^ PAR, which may have been adequate in some species to allow photosynthetic fixation of any CO_2_ generated. C_2_H_4_ is a plant signalling molecule, so it is not surprising to detect it during our experiments, but these are net emission rates, suggesting that exposure to UV radiation increases the C_2_H_4_ emission rate. In 93% of the species, the C_2_H_4_ emission rate from control leaves (no UV) accounted for <42% of C_2_H_4_ released; in 60% of species, the proportion was as low as <20% of the UV‐induced output. Work by McLeod *et al*. (2008), using pure plant pectin as an analogue, suggested that plants are also likely to emit longer chain alkanes (C2 and C3) as well as CH_4_. Here we report evidence for a net increase in C_3_H_8_ with exposure to UV being observed in all species investigated.

### Taxonomic and functional groupings of species

The consideration of CH_4_ emission rates in relation to plant functional types and taxonomic classification (Fig. 4) illustrates some possible differences between plant groups. Different rates of CH_4_ and other gaseous emissions might be expected to be due to differences in leaf chemical composition, leaf absorbance and reflectance, leaf structural characteristics, cuticle thickness, ability to produce UV‐protective pigments and efficiency of ROS scavenging. Leaf pectin content varies across species and significantly between taxonomic groups (O'Neill & York 2003), thus, if pectin is a significant source within leaves, CH_4_ emission is also likely to vary greatly between taxonomic groups. The responses of plant functional types to increased UV‐B radiation was reviewed by Gwynn‐Jones *et al*. (1999). They concluded that bryophytes are most sensitive to increases in UV‐B while tree species are least sensitive and that this could be related to their potential for producing UV‐B absorbing compounds. Our study did not include bryophytes and our data do not provide sufficient replicates of sedge and fern to warrant firm conclusions. However, the CH_4_ emissions from the 17 tree species formed the lowest group (Fig. 4a) and were therefore consistent with the conclusions of Gwynn‐Jones *et al*. (1999). Day *et al*. (1993) suggested that a considerable proportion of UV‐B penetrates beyond the epidermis, particularly in herbaceous species and this is consistent with our observation (Fig. 4a) that herbs showed a higher range of CH_4_ emission rates than other groups. The single fern studied was the highest emitter of CH_4_, followed by grasses, shrubs, trees and finally, the single sedge species tested. This order of emission rates may relate to the growth habit of the plants types involved. Trees generally form the upper storey of a habitat and may have evolved more effective mechanisms of UV protection, while shrubs and herbaceous plants often occupy the understorey, where they may experience some protection from UV exposure due to shading effects of the overlying canopy and thus develop fewer UV‐protective properties (Fraser *et al*. 2011). Notably, ferns often populate heavily shaded understorey habitats, whereas grasses and sedges are often found growing in open UV‐exposed habitats. An alternative grouping based on divisions of the Plantae kingdom: Dicotyledoneae, Monocotyledoneae, Pinopsida and Polypodiopsida (Fig. 4b) suggests that dicotyledons and Pinopsida may be lower emitters of CH_4_ under UV irradiation than the monocotyledons. The four monocotyledons with the lowest CH_4_ emissions (*Carex rostrata* Stokes, *Phyllostachys aurea* Rivière & C. Rivière, *Saccharum officinarum* L. and *Deschampsia cespitosa* P. Beauv.) are members of the Cyperaceae and Poaceae, which are classified by the Angiosperm Phylogeny Group III within the Cyperales (Stevens 2012). The Cyperales are notably low in pectin, whereas most other Monocotyledoneae are much richer in pectin (Jarvis *et al*. 1988). Thus, the low CH_4_ emissions observed in the Cyperales support the possibility that the higher CH_4_ emissions seen in the other monocotyledons are due their higher pectin content.

Our observed correlation of CH_4_ emissions with epidermal absorbance and lignin content, and the possible differences between plants grown inside a glasshouse or outside (Fig. 3), highlights the importance of the prior radiation environment (both UV and PAR) of the plant material studied. Differences in structure and physiology between ‘shade’ and ‘sun’ leaves are well known (Gwynn‐Jones *et al*. 1999) and are also likely to affect UV‐induced gaseous emissions from foliage. Such differences between plant source materials may explain some of the variability between emission rates reported in the literature in addition to differences in UV irradiances, UV sources and spectral composition. Future studies should ideally report the radiation and growing conditions of the plant material in full and where appropriate measure plants grown in their natural radiation environment.

## Conclusions

Our data strongly suggest that the terrestrial vegetation sampled, including trees, shrubs, herbs, grasses, a sedge and a fern, can emit CH_4_, CO, C_2_H_4_ and C_3_H_8_ from leaves when exposed to spectrally weighted UV levels equivalent to ambient levels of UV observed outside. Our experimental method suggests that these emissions originate within the foliage and do not result from an inflow of water containing dissolved CH_4_ in the transpiration stream. They corroborate the observations of other studies that have previously reported foliar emissions of CH_4_ under aerobic conditions (Keppler *et al*. 2006; McLeod *et al*. 2008; Vigano *et al*. 2008; Bruhn *et al*. 2009) and they do not suggest a need to revise the estimate of Bloom *et al*. (2010) that UV‐driven CH_4_ emissions from foliage contribute <0.2% of global CH_4_ sources.

The rate of UV‐driven CH_4_ emission from leaves was influenced by their epidermal UV absorbance and lignin content and is therefore likely to vary between species, not only because of their inherent structural and metabolic differences but also because of the plant growing conditions and UV and PAR environment. Consequently, one would expect the same species to show variable rates of UV‐driven CH_4_ emission, whether grown inside a controlled environment facility without UV exposure, inside a glasshouse with exposure to some UV‐A or outside where shading by other vegetation may greatly modify prior UV exposure. Future studies that use UV‐driven foliar emission rates in global upscaling calculations should therefore be based upon measurements that are relevant to plants in their natural environments as well as on the use of appropriate irradiance levels and spectral distribution of UV radiation.

## Supporting information


**Figure S1.** View of the UV irradiation equipment showing Q‐Panel UV‐313 fluorescent lamps, four chambers with quartz windows and water cooling, gas syringes for sampling, UV and PAR sensors.
**Table S1.** Net UV‐induced gaseous emissions of CH_4,_ CO, CO_2,_ C_2_H_4,_ C_2_H_6_ and C_3_H_8_ from plant leaves, expressed per unit leaf dry weight and per unit leaf area, when irradiated with 7.1 W m^−2^ (CH_4_‐weighted) ultraviolet radiation (280–400 nm) from Q‐Panel UV313 fluorescent lamps filtered with 125 *μ* m cellulose diacetate at 25 °C. Plants were grown inside a glasshouse (GH) of the Royal Botanic Gardens, Edinburgh with appropriate temperature and humidity control for the species or outside (O).
