# Supplementary material for: Emission of methane, carbon monoxide, carbon dioxide and short‐chain hydrocarbons from vegetation foliage under ultraviolet irradiation
Source: Plant Cell Environ. 2015 Jan 23;38(5):980–9. doi: 10.1111/pce.12489 (PMC4964915; doi:10.1111/pce.12489)

Figure S1. View of the UV irradiation equipment showing Q-Panel UV-313 fluorescent lamps, four chambers with quartz windows and water cooling, gas syringes for sampling, UV and PAR sensors.


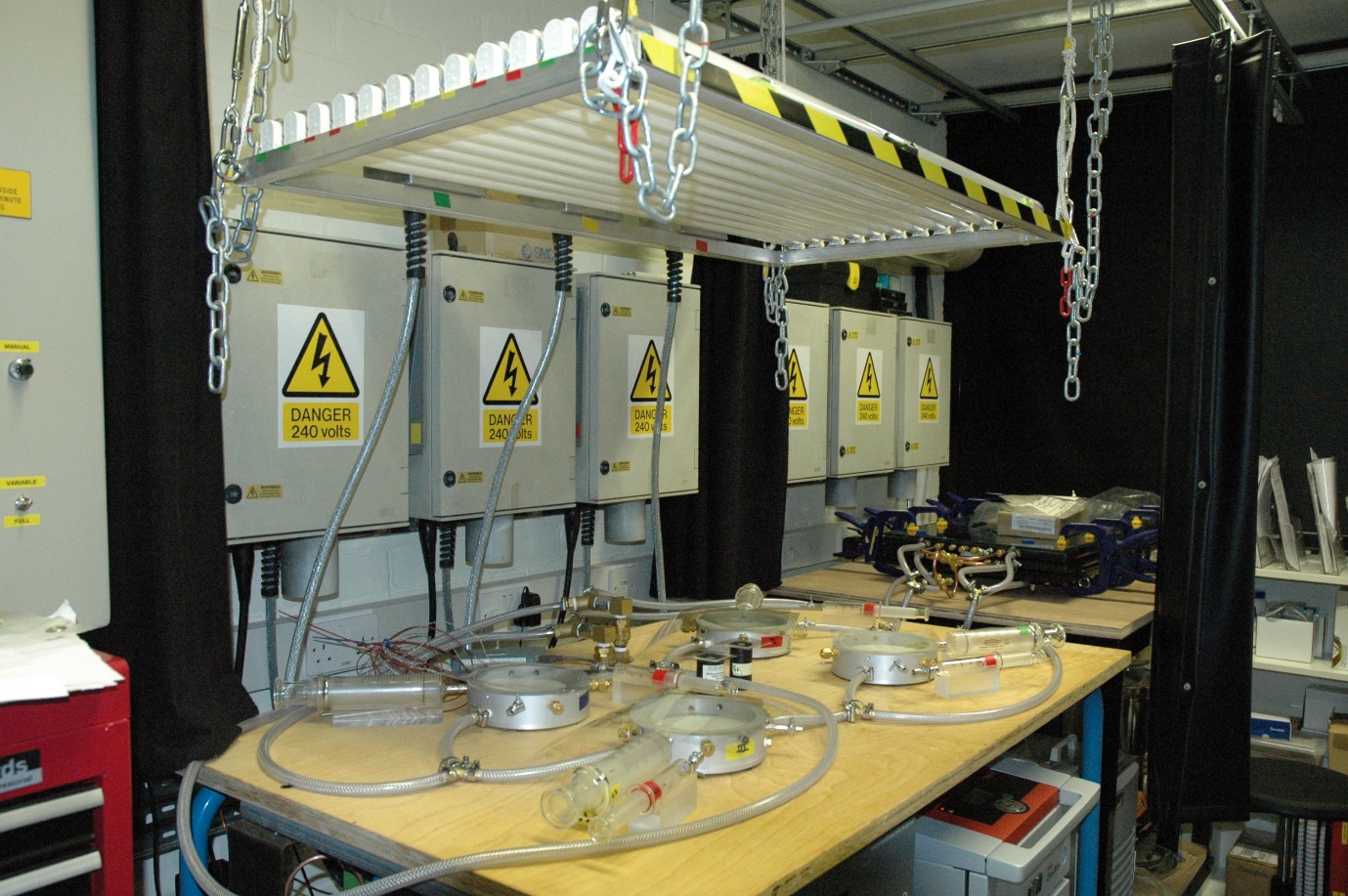

Supplement: Supplementary file 1 — Figure S1. View of the UV irradiation equipment showing Q‐Panel UV‐313 fluorescent lamps, four chambers with quartz windows and water cooling, gas syringes for sampling, UV and PAR sensors. Table S1. Net UV‐induced gaseous emissions of CH4, CO, CO2, C2H4, C2H6 and C3H8 from plant leaves, expressed per unit leaf dry weight and per unit leaf area, when irradiated with 7.1 W m−2 (CH4‐weighted) ultraviolet radiation (280–400 nm) from Q‐Panel UV313 fluorescent lamps filtered with 125 μ m cellulose diacetate at 25 °C. Plants were grown inside a glasshouse (GH) of the Royal Botanic Gardens, Edinburgh with appropriate temperature and humidity control for the species or outside (O). [file PCE-38-980-s001.zip › PCE_12489_Supporting Figure S1.docx]
