# Supplementary material for: Emission of methane, carbon monoxide, carbon dioxide and short‐chain hydrocarbons from vegetation foliage under ultraviolet irradiation
Source: Plant Cell Environ. 2015 Jan 23;38(5):980–9. doi: 10.1111/pce.12489 (PMC4964915; doi:10.1111/pce.12489)
Supplement: Supplementary file 1 — Figure S1. View of the UV irradiation equipment showing Q‐Panel UV‐313 fluorescent lamps, four chambers with quartz windows and water cooling, gas syringes for sampling, UV and PAR sensors. Table S1. Net UV‐induced gaseous emissions of CH4, CO, CO2, C2H4, C2H6 and C3H8 from plant leaves, expressed per unit leaf dry weight and per unit leaf area, when irradiated with 7.1 W m−2 (CH4‐weighted) ultraviolet radiation (280–400 nm) from Q‐Panel UV313 fluorescent lamps filtered with 125 μ m cellulose diacetate at 25 °C. Plants were grown inside a glasshouse (GH) of the Royal Botanic Gardens, Edinburgh with appropriate temperature and humidity control for the species or outside (O). [file PCE-38-980-s001.zip › PCE_12489_Supporting Table S1.docx]

**Table S1.** Net UV-induced gaseous emissions of CH_4,_ CO, CO_2,_ C_2_H_4,_ C_2_H_6_ and C_3_H_8_ from plant leaves, expressed per unit leaf dry weight and per unit leaf area, when irradiated with 7.1 W m^-2^ (CH_4_-weighted) ultraviolet radiation (280-400 nm) from Q-Panel UV313 fluorescent lamps filtered with 125 µm cellulose diacetate at 25^o^C. Plants were grown inside a glasshouse (GH) of the Royal Botanic Gardens, Edinburgh with appropriate temperature and humidity control for the species or outside (O).

| **GH/O ^a^** | **Species** | **Classification** | **Group** | **Net emissions (ng g^-1^ DW leaf h^-1^) ^b^** | | | | | |  | **Net emissions (ng cm^-2^ h^-1^) ^c^** | | | | | |
| --- | --- | --- | --- | --- | --- | --- | --- | --- | --- | --- | --- | --- | --- | --- | --- | --- |
|  |  |  |  | **CO** | **CH_4_** | **CO_2_** | **C_2_H_4_** | **C_2_H_6_** | **C_3_H_8_** |  | **CO** | **CH_4_** | **CO_2_** | **C_2_H_4_** | **C_2_H_6_** | **C_3_H_8_** |
| GH | *Brownea grandiceps* Jacq. | Dicotyledoneae | Tree | 69.2 | 5.9 | 1.6 x 10^5^ | 13.2 | -1.4 | 7.3 |  | 0.28 | 0.04 | 1.17 x 10^3^ | 0.07 | -0.01 | 0.05 |
| O | *Gossypium arboreum* L. | Dicotyledoneae | Tree | -72.5 | 6.4 | 4.8 x 10^5^ | 33.2 | -1.2 | 44.8 |  | -0.40 | 0.03 | 2.98 x 10^3^ | 0.17 | -0.01 | 0.23 |
| GH | *Pseudobombax longiflorum* (Mart. & Zucc.) A.Robyns | Dicotyledoneae | Tree | 114.0 | 5.6 | -4.8 x 10^4^ | 14.7 | 0.6 | 17.3 |  | 0.72 | 0.03 | -6.51 x 10^2^ | 0.08 | <0.01 | 0.10 |
| GH | *Tillandsia usneoides* (L.) L. | Monocotyledoneae | Herb | 341.0 | 24.1 | 8.3 x 10^5^ | 59.6 | 2.3 | 100.4 |  | 3.06 | 0.22 | 7.01 x 10^3^ | 0.53 | 0.02 | 0.91 |
| GH | *Saccharum officinarum* L. | Monocotyledoneae | Grass | 197.8 | 8.8 | -2.3 x 10^5^ | 17.3 | 0.7 | 22.6 |  | 0.82 | 0.04 | -1.66 x 10^3^ | 0.06 | <0.01 | 0.08 |
| GH | *Crinum asiaticum* L. | Monocotyledoneae | Herb | 89.6 | 12.3 | -2.6 x 10^5^ | 11.6 | 0.4 | 20.8 |  | 0.44 | 0.06 | -1.38 x 10^3^ | 0.06 | <0.01 | 0.11 |
| GH | *Musa acuminata* Colla | Monocotyledoneae | Herb (banana) | 553.0 | 14.9 | -4.5 x 10^5^ | 123.0 | -0.2 | 99.6 |  | 1.98 | 0.05 | -1.65 x 10^3^ | 0.44 | <0.01 | 0.36 |
| GH | *Ficus benjamina* L. | Dicotyledoneae | Tree | 73.8 | 3.9 | 1.2 x 10^5^ | 13.9 | -0.7 | 12.9 |  | 0.38 | 0.02 | 6.75 x 10^2^ | 0.07 | <0.01 | 0.07 |
| O | *Rhododendron ponticum - Iberian form* L. | Dicotyledoneae | Shrub | -0.3 | 4.4 | 5.4 x 10^4^ | 14.3 | 0.3 | 16.4 |  | -0.02 | 0.08 | -2.82 x 10^2^ | 0.25 | <0.01 | 0.28 |
| O | *Rosmarinus officinalis* L. | Dicotyledoneae | Shrub | 74.4 | 5.0 | 2.7 x 10^5^ | 26.1 | -0.9 | 34.0 |  | 1.46 | 0.10 | 5.75 x 10^3^ | 0.52 | -0.02 | 0.68 |
| GH | *Hevea brasiliensis* (Willd. ex A.Juss.) Müll.Arg. | Dicotyledoneae | Tree | 252.3 | 1.0 | -3.8 x 10^5^ | 7.8 | -0.5 | 5.0 |  | 1.34 | 0.01 | -1.78 x 10^3^ | 0.04 | <0.01 | 0.03 |
| GH | *Coccoloba pubescens* L. | Dicotyledoneae | Tree | 67.3 | 2.5 | -2.5 x 10^4^ | 4.7 | 0.3 | 5.7 |  | 0.73 | 0.03 | -3.13 x 10^2^ | 0.05 | <0.01 | 0.06 |
| O | *Chamaecyparis obtusa* (Siebold & Zucc.) Endl. | Pinopsida | Tree | 103.9 | 4.5 | 6.6 x 10^3^ | 13.5 | 0.2 | 19.0 |  | 2.41 | 0.10 | -6.75x 10^2^ | 0.29 | <0.01 | 0.40 |
| O | *Pinus ponderosa* Douglas ex C.Lawson | Pinopsida | Tree | 81.4 | 1.2 | 1.4 x 10^4^ | 15.1 | -0.3 | 14.8 |  | 1.63 | 0.02 | 3.24 x 10^2^ | 0.30 | -0.01 | 0.30 |
| O | *Deschampsia cespitosa* P.Beauv. | Monocotyledoneae | Grass | 500.8 | 9.8 | -8.7 x 10^5^ | 38.0 | 1.0 | 105.5 |  | 4.75 | 0.10 | -3.39 x 10^3^ | 0.46 | 0.01 | 1.17 |
| O | *Salvia officinalis* L. | Dicotyledoneae | Shrub | 211.2 | 17.9 | 2.7 x 10^5^ | 39.4 | 1.2 | 137.0 |  | 1.08 | 0.11 | 4.83 x 10^3^ | 0.24 | 0.01 | 0.81 |
| GH | *Cecropia peltata* L. | Dicotyledoneae | Tree | -37.8 | 5.7 | -2.4 x 10^6^ | -97.2 | 0.4 | 24.1 |  | -0.14 | 0.02 | -1.12 x 10^4^ | -0.42 | <0.01 | 0.08 |
| GH | *Herrania nycterodendron* R.E.Schult. | Dicotyledoneae | Tree | 162.6 | 8.7 | -1.5 x 10^5^ | 21.4 | -0.7 | 20.7 |  | 1.00 | 0.05 | -1.02 x 10^3^ | 0.13 | <0.01 | 0.12 |
| O | *Phyllostachys aurea* Rivière & C.Rivière | Monocotyledoneae | Grass (bamboo) | 480.8 | 7.7 | 1.4 x10^4^ | 39.8 | 1.2 | 59.5 |  | 2.05 | 0.03 | 8.56 x 10^2^ | 0.19 | 0.01 | 0.28 |
| O | *Carex rostrata* Stokes | Monocotyledoneae | Sedge | 197.7 | 4.5 | 2.5 x 10^5^ | 37.8 | 0.3 | 40.6 |  | 0.61 | 0.01 | 7.04 x 10^2^ | 0.11 | <0.01 | 0.12 |
| GH | *Aspidosperma tomentosum* Mart. | Dicotyledoneae | Tree | 175.7 | 3.3 | -1.7 x 10^5^ | 8.9 | 0.2 | 20.7 |  | 0.83 | 0.02 | -8.55 x 10^2^ | 0.04 | <0.01 | 0.10 |
| GH | *Anacardium occidentale* L. | Dicotyledoneae | Tree | 138.4 | 2.1 | 1.9 x 10^6^ | 36.3 | 1.9 | 9.3 |  | 0.90 | 0.01 | 7.61 x 10^3^ | 0.17 | 0.01 | 0.04 |
| GH | *Physocalymma scaberrimum* Pohl | Dicotyledoneae | Tree | 226.2 | 12.0 | 3.7 x 10^4^ | 12.2 | -0.3 | 14.2 |  | 0.84 | 0.05 | 1.89 x 10^2^ | 0.05 | <0.01 | 0.05 |
| GH | *Lafoensia pacari* A.St.-Hil. | Dicotyledoneae | Tree | 34.0 | 1.7 | 1.9 x 10^5^ | 14.1 | -0.4 | 11.0 |  | 0.30 | 0.01 | 8.22 x 10^2^ | 0.09 | <0.01 | 0.07 |
| GH | *Zingiber officinale* Roscoe | Monocotyledoneae | Herb | 498.1 | 23.5 | 6.6 x 10^5^ | 131.7 | -0.2 | 100.6 |  | 0.98 | 0.05 | 1.23 x 10^3^ | 0.27 | <0.01 | 0.19 |
| GH | *Curcuma longa* L. | Monocotyledoneae | Herb | -218.5 | 17.4 | -8.1 x 10^4^ | 52.0 | 7.6 | 69.0 |  | -0.15 | 0.02 | -2.45 x 10^2^ | 0.08 | 0.01 | 0.10 |
| GH | *Nephrolepsis exaltata* (L.) Schott | Polypodiopsida | Fern | 807.6 | 31.8 | 7.6 x 10^4^ | 91.8 | -0.4 | 123.3 |  | 1.54 | 0.06 | 2.07 x 10^2^ | 0.17 | <0.01 | 0.23 |
| GH | *Cybistax antisyphilitica* (Mart.) Mart. | Dicotyledoneae | Tree | 185.6 | 9.7 | 1.9 x 10^5^ | 47.8 | 0.3 | 32.1 |  | 1.02 | 0.05 | 1.38 x 10^3^ | 0.27 | <0.01 | 0.18 |
| O | *Metasequoia glyptostroboides* Hu & W.C.Cheng | Pinopsida | Tree | -101.1 | 1.9 | 2.1 x 10^5^ | 13.4 | 0.0 | 30.5 |  | -0.84 | 0.02 | 2.36 x 10^3^ | 0.12 | <0.01 | 0.26 |
| O | *Fagus sylvatica* L. | Dicotyledoneae | Tree | 100.0 | 0.2 | -1.5 x 10^4^ | 94.2 | 0.2 | 7.7 |  | 0.81 | <0.01 | 1.93 x 10^3^ | 0.79 | <0.01 | 0.06 |
| ^a^ GH/O = plants were either grown in a glasshouse (GH) or outside (O). | | | |  |  |  |  |  |  |  |  |  |  |  |  |  |
| ^b^ DW = Dry weight of leaves after 48 h drying at 50^o^C. | | |  |  |  |  |  |  |  |  |  |  |  |  |  |  |
| ^c^ Leaf area was electronically determined using a flatbed scanner and analysed with ImageJ software (Abramoff *et al.* "Image Processing with ImageJ" ,Biophotonics International (2004) 11 (7) : 36-42. | | | | | | | | | | | | | | | | |
